# National and sub-national variation in patterns of febrile case management in sub-Saharan Africa

**DOI:** 10.1038/s41467-018-07536-9

**Published:** 2018-11-26

**Authors:** Victor A. Alegana, Joseph Maina, Paul O. Ouma, Peter M. Macharia, Jim Wright, Peter M. Atkinson, Emelda A. Okiro, Robert W. Snow, Andrew J. Tatem

**Affiliations:** 1 Geography and Environmental Science, University of Southampton, Southampton, SO17 1BJ UK; 2 Population Health Theme, Kenya Medical Research Institute-Wellcome Trust Research Programme, Nairobi, Kenya; 3 Faculty of Science and Technology, Lancaster University, Lancaster, LA1 4YW UK; 4 School of Natural and Built Environment, Queen’s University Belfast, Belfast, BT7 1NN UK; 5 Institute of Geographical Sciences and Natural Resources Research, Chinese Academy of Sciences, A11 Datun Road, Beijing, 100101 China; 6 Centre for Tropical Medicine and Global Health, Nuffield Department of Clinical Medicine, University of Oxford, Oxford, OX3 7LJ UK; 7 Flowminder Foundation, Stockholm, SE-11355 Sweden

## Abstract

Given national healthcare coverage gaps, understanding treatment-seeking behaviour for fever is crucial for the management of childhood illness and to reduce deaths. Here, we conduct a modelling study triangulating household survey data for fever in children under the age of five years with georeferenced public health facility databases (*n* = 86,442 facilities) in 29 countries across sub-Saharan Africa, to estimate the probability of seeking treatment for fever at public facilities. A Bayesian item response theory framework is used to estimate this probability based on reported fever episodes, treatment choice, residence, and estimated travel-time to the nearest public-sector health facility. Findings show inter- and intra-country variation, with the likelihood of seeking treatment for fever less than 50% in 16 countries. Results highlight the need to invest in public healthcare and related databases. The variation in public sector use illustrates the need to include such modelling in future infectious disease burden estimation.

## Abstract (web summary)

Treatment-seeking for fever is widely used to estimate treatment of childhood infections, but cross-country comparisons are problematic. Here, the authors estimate the probability of seeking treatment for fever at public facilities across 29 countries by quantifying person-level latent variables.

## Introduction

Febrile illness, despite being associated with several childhood illnesses^1–3^, is a poorly understood feature of infectious disease burden in sub-Saharan Africa. Importantly, intensity of infection shapes the course of illness^4,5^, leading to variation in the perception of disease as well as actions taken for treatment in public health systems or privately^6^. This variation is key to understanding treatment-seeking for childhood illness in the public health sectors across sub-Saharan Africa (SSA).

Treatment-seeking for fever, as measured in current nationally representative household surveys, is presently the most widespread surrogate method of estimating treatment of most suspected childhood infectious diseases. However, despite previous descriptive studies on treatment-seeking for fever^6–9^, variation in the interpretation of what constitutes fever at individual level, driven collectively by disease factors, perception of severity and health system factors, makes comparisons problematic. Therefore, there is a need for increased understanding of individual characteristics related to seeking treatment for fever within health systems in SSA, specifically on the determinants of treatment-seeking behaviour and the mechanisms underpinning variation at national and sub-national levels. Modelling treatment-seeking health behaviour would support future case burden estimation at peripheral health facilities and, in turn, improve planning of appropriate peripheral health facilities^3,10,11^.

Differences in the interpretation of fever and perception of its severity affect treatment-seeking behaviour. Person-level latent characteristics concerning ability to seek treatment should form the backbone of understanding collective population health behaviour. However, previous studies did not examine latent characteristics that are related to such person-level interpretations from surveys across different cultures, thus, preventing quantitative comparison at the national level. The main objectives of this study were to conduct such a quantitative analysis from cross-sectional surveys and to translate person-latent effects into population-level probabilistic estimates that are comparable at national or sub-national level. Therefore, no attempt was made to quantify fever attributable to malaria, for example, as recently estimated here^12^. Bayesian item response theory (IRT)^13^ was employed to quantify person-level characteristics, namely the latent ability to seek treatment at the nearest public health facility, adjusting for travel time and residence. These latent characteristics were then used to estimate the probability of seeking treatment for fever in the public sector based on geography.

## Results

### Summary of data on fever prevalence

In total, 407,218 children under the age of 5 years were included using data from recent nationally representative household surveys in 43 sub-Saharan countries (household survey data summarised in Supplementary Data 1). Of the 407,218 children included, ~99,613 reported a fever episode 2weeks prior to the survey and, of these, 56,719 (56.9%) sought treatment in the public sector. Prevalence of fever varied within and between countries and was highest in Nigeria with a posterior median of 44.2% (interquartile range 33.3–48.6%). Other notable country-level prevalences were: Burundi 41.7% (34.8–46.2%), Kenya 38.5% (29.8–44.4%), Ethiopia 13.9% (8.4 – 16.0%), The Gambia 7.0% (6.2–9.7%) and Djibouti 4.8% (3.3–6.25%).

**Fig. 1:**
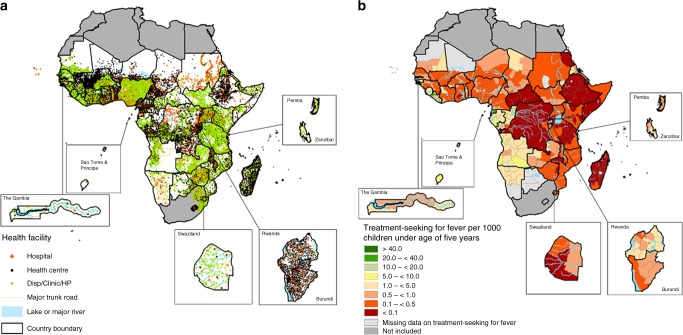
Health facilities and cases of fever. **a** The distribution of public health facilities in sub-Saharan Africa by type. **b** Aggregate estimates of cases of fever treated at Administrative level 1 per 1000 children under five (weighted by the population at risk). Overall, 56,719 out of 99,631 sampled children sought fever treatment in the public sector. Data assemblies were between 2010–2016, except for South Sudan and Djibouti. Northern Africa countries and South Africa were not included in the analysis. Data were obtained from national reports in countries where georeferenced data were not available, for example, Somalia

Fig. 1a shows the distribution of health facilities while Fig. 1b shows the pattern of population-weighted, observed treatment-seeking for fever at a public health facility at the sub-national level in sub-Saharan Africa. The rate of treatment-seeking for fever was low in the Democratic Republic of the Congo, Ethiopia, Southern Somalia, Central African Republic and Madagascar where the aggregated national estimate in the public sector was less than 0.1 per 1000 under 5 children (Fig. 1b). Aggregated national estimates exceeded 10 per 1000 children in parts of Equatorial Guinea and Gabon. Although Fig. 1b exhibits heterogeneity at the country-level, these aggregation-based estimates are challenging to interpret and compare across population groups due to differences in perceptions of fever across different populations in sub-Saharan Africa. It was, therefore, necessary to estimate person-level latent traits related to observed data and use these to provide probabilistic estimates at the national or sub-national level.

### Summary of assembled public health facilities

**Fig. 2:**
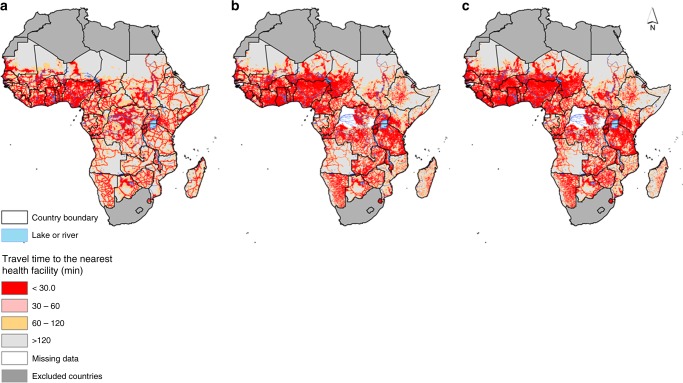
Travel time maps. Travel time to the nearest health facilities at 1 km spatial resolution in sub-Saharan Africa based on combined motorised transport and walking, adjusting for topography for **a** major general hospitals, **b** health centres and **c** dispensaries, clinics and health posts (lower-tier health facilities). The white patches are regions where health centre or dispensary maps were unavailable (the Congo, Equatorial Guinea, Guinea Bissau and two regions in the Democratic Republic of Congo (DRC))

Overall, 86,442 public-based health facilities were assembled in SSA (Fig. 1a). 5250 (6.3%) of these comprised major regional and district hospitals, while the rest constituted health centres (22.2%) or dispensaries (71.5%). The largest number of facilities were in Nigeria, Tanzania and Kenya. Figure 2 (a–c) shows geographic accessibility to hospitals, health centres and lower-tier health facilities (dispensaries, clinic or health posts) based on travel time. Geographic access varied based on density of public health facilities and was overall congruent to population density.

### Probability of seeking fever treatment in the public sector

**Fig. 3:**
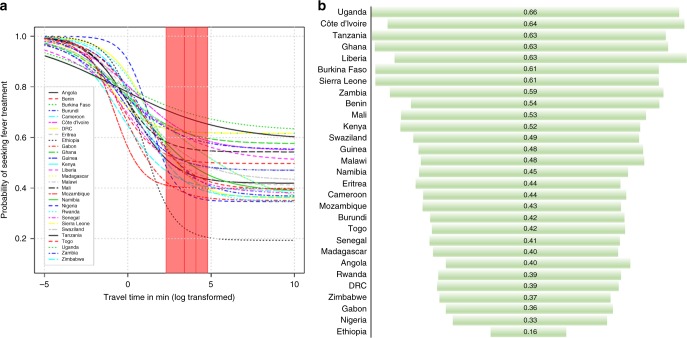
Probability of seeking fever treatment. **a** National variation in the probability of seeking fever treatment (posterior median) for any type of public health facility. The red shaded region and vertical lines represent the probability of seeking treatment for fever in the public sector by travel time at 10 min, 30 min, 1 h and 2 h. **b** Funnel plot representing national posterior median probability of seeking fever treatment in the public sector (length of bar indicates the probability) at 2 h’ travel time to the nearest primary healthcare facility (dispensary, clinic or health post). The probability of treatment was modelled based on the item response theory (IRT) model

Fig. 3a shows national variation in the probability of treatment-seeking for fever by travel time to the nearest public healthcare facility (Supplementary Figure 1 shows spatial variation by facility type). These patterns were estimated from the posterior medians of latent parameters based on Bayesian item response theory modelling. The shaded region and vertical lines of Fig. 3a show the probability of seeking treatment for fever in the public sector by travel time at 10 min, 30 min, 1 h and 2 h. The posterior median summaries for these time intervals are presented in Table 1.

**Table 1:** National-level modelled estimates of the probability of seeking treatment for fever in the public sector (Posterior median and the 95% Bayesian credible interval) by travel time, for the shaded region in Fig. 3a, in 29 countries

Country	Health facilities	Clusters (Georeferenced)	10 min	30 min	1 h	2 h
Angola	1511	238 (238)	0.52 (0.37–0.54)	0.46 (0.34– 0.53)	0.45 (0.32–0.53)	0.43 (0.31–0.53)
Benin	823	750 (750)	0.53 (0.29– 0.68)	0.51 (0.27– 0.67)	0.5 (0.26–0.67)	0.5 (0.25–0.67)
Burkina Faso	1782	252 (252)	0.63 (0.32– 0.79)	0.62 (0.31– 0.79)	0.62 (0.31– 0.79)	0.62 (0.3–0.79)
Burundi	743	200 (200)	0.56 (0.42– 0.58)	0.47 (0.4–0.55)	0.44 (0.38–0.55)	0.41 (0.37– 0.55)
Cameroon	3061	578 (578)	0.47 (0.28– 0.55)	0.43 (0.26–0.54)	0.41 (0.25– 0.54)	0.41 (0.24– 0.54)
Côte d’Ivoire	1816	351 (351)	0.67 (0.44– 0.74)	0.62 (0.43– 0.73)	0.6 (0.43–0.73)	0.59 (0.43–0.73)
DRC	6241	536 (536)	0.57 (0.5–0.55)	0.46 (0.47–0.52)	0.42 (0.46– 0.52)	0.4 (0.44–0.52)
Eritrea	270	86 (86)	0.55 (0.34– 0.62)	0.49 (0.34–0.6)	0.46 (0.33–0.6)	0.44 (0.33–0.6)
Ethiopia	902	596 (571)	0.33 (0.35–0.28)	0.24 (0.27– 0.26)	0.22 (0.23– 0.26)	0.21 (0.2–0.26)
Gabon	542	334 (334)	0.42 (0.28–0.5)	0.38 (0.25–0.5)	0.37 (0.23–0.5)	0.36 (0.22–0.5)
Ghana	1960	353 (349)	0.65 (0.41–0.73)	0.62 (0.41– 0.72)	0.61 (0.41– 0.72)	0.6 (0.41–0.72)
Guinea	1172	300 (300)	0.54 (0.33– 0.63)	0.51 (0.31–0.62)	0.49 (0.29– 0.62)	0.48 (0.29– 0.62)
Kenya	7149	1829 (1838)	0.64 (0.38– 0.63)	0.59 (0.37– 0.59)	0.56 (0.37–0.59)	0.52 (0.37– 0.59)
Liberia	594	472 (472)	0.64 (0.4–0.71)	0.6 (0.4 –0.68)	0.58 (0.39– 0.68)	0.57 (0.39– 0.68)
Madagascar	2657	284 (284)	0.64 (0.38–0.63)	0.59 (0.37–0.59)	0.56 (0.37– 0.59)	0.52 (0.37– 0.59)
Malawi	561	140 (140)	0.53 (0.37– 0.63)	0.5 (0.36–0.62)	0.48 (0.35– 0.62)	0.48 (0.34– 0.62)
Mali	1350	590 (413)	0.58 (0.35–0.7)	0.56 (0.33–0.7)	0.55 (0.32– 0.69)	0.55 (0.32–0.69)
Mozambique	1175	610 (609)	0.41 (0.22– 0.55)	0.4 (0.21–0.54)	0.4 (0.21–0.54)	0.4 (0.2–0.54)
Namibia	461	407 (407)	0.58 (0.38– 0.62)	0.51 (0.38–0.59)	0.48 (0.38– 0.58)	0.45 (0.37– 0.58)
Nigeria	20,903	567 (562)	0.48 (0.45– 0.44)	0.38 (0.36– 0.42)	0.36 (0.31–0.42)	0.35 (0.27– 0.42)
Rwanda	498	492 (492)	0.57 (0.47– 0.57)	0.47 (0.45–0.53)	0.42 (0.44– 0.53)	0.39 (0.43– 0.53)
Senegal	1604	706 (705)	0.51 (0.32– 0.55)	0.46 (0.3– 0.53)	0.43 (0.29– 0.53)	0.42 (0.28– 0.53)
Sierra Leone	1351	435 (435)	0.63 (0.32– 0.79)	0.62 (0.31– 0.79)	0.62 (0.31–0.79)	0.62 (0.3–0.79)
Swaziland	146	27 (27)	0.59 (0.36– 0.68)	0.54 (0.36–0.66)	0.51 (0.36– 0.66)	0.49 (0.36– 0.66)
Tanzania	11,652	330 (330)	0.71 (0.42– 0.75)	0.68 (0.42– 0.73)	0.67 (0.42– 0.72)	0.65 (0.42– 0.72)
Togo	246	210 (210)	0.55 (0.39– 0.59)	0.48 (0.36– 0.56)	0.45 (0.35– 0.56)	0.43 (0.33–0.56)
Uganda	3936	806 (806)	0.73 (0.45– 0.78)	0.7 (0.45–0.76)	0.69 (0.45– 0.75)	0.68 (0.45– 0.75)
Zambia	1345	721 (721)	0.64 (0.39– 0.72)	0.6 (0.38–0.7)	0.59 (0.38–0.7)	0.58 (0.37–0.7)
Zimbabwe	1444	406 (406)	0.5 (0.39– 0.52)	0.43 (0.35–0.5)	0.4 (0.33–0.5)	0.39 (0.31–0.5)

The probability of treatment was estimated based on any type of facility and was lowest in Ethiopia and highest in Uganda

Fig. 3b shows a funnel plot of national variation in the probability of treatment-seeking for fever at the nearest primary healthcare facility (dispensaries, clinics or health posts). Whilst there was variation at the national and sub-national levels, modelled population probability of seeking treatment for fever at the nearest primary healthcare facility within 30 min’ travel time was low in Ethiopia (0.21, 95% Bayesian credible intervals 0.16–0.23) and Nigeria (0.37, 0.34– 0.42). In Uganda and Tanzania there was respectively a 69% [0.69 (0.44–0.76)] and 66% [0.66 (0.43–0.72)] chance of seeking treatment at 30 min’ travel time to the nearest primary public healthcare facility, although the credible intervals were wider (Supplementary Data 2).

The strength of distance-decay effect varied at the country-level and was minimal in some countries (e.g., Tanzania, Burkina Faso) and varied more in Gabon, Nigeria, and Ethiopia. Overall, the effect suggested a reduction in the proportion of children seeking treatment at greater travel time. The minimum threshold probability of seeking treatment for fever at 2 h was 0.21 in Ethiopia (95% Bayesian Credible Interval 0.20–0.26 black dashed line) and Nigeria 0.35 (0.27–0.42), suggesting at most a 21 and 35% chance of treatment for people living more than 2 h from a facility, respectively.

Table 1 summarises posterior median national-level treatment-seeking for fever probability estimates at 10, 30, 60 and 120 min time intervals to any public health facility (i.e., the shaded region of Fig. 3a, which shows national variation). Maps of sub-national variation are shown in Supplementary Figure 1. In 16 of the 29 countries, the posterior median (probability of seeking fever treatment) was less than 50% at 2 h’ travel time from the nearest facility. For example, the posterior median probability at 2 h’ travel time for Madagascar was 0.49 (0.36–0.66), suggesting ~50% of fever cases were not treated in the public sector at 2 h’ travel time.

## Discussion

The probability of obtaining appropriate treatment for febrile illnesses is a key factor in reducing disease morbidity and mortality in SSA. Using a combination of health facility databases and household level reported actions concerning the treatment of fever, our findings show national and sub-national variation in the probability of seeking treatment for fever in the public sector. From the 29 countries examined, the probability of treatment-seeking for children living 2 h from a public health facility was less than 50% in 16 countries, which suggested that in these countries, at least half of febrile cases were untreated or treated outside the public sector. There was a marginally higher likelihood of fever treatment-seeking at a primary healthcare facility (dispensary or clinic) compared with a major hospital. The novelty of this study was in translating latent characteristics related to treatment-seeking choices at individual level to probabilistic metrics, so as to aid national and sub-national comparability. The findings are, therefore, not only useful in understanding patterns of service delivery, geographic coverage gaps and utilisation, but also are applicable to future disease burden estimation.

Health facility spatial databases form a bedrock for many health studies. A triangulation of these healthcare information system data with household surveys provides an insight into public health system failures. It is not clear if estimates on treatment-seeking quantified here for children under the age of five reflect treatment-seeking for childhood diseases with fever symptoms in the public sector or in other age-groups. With declining malaria prevalence, recent evidence suggests most fever cases in children under the age of 5 years are not due to malaria infection^12^. Fever, in general, is known to be associated with other childhood illnesses including, but not limited to, bacterial infections, parasitic infections, and upper or lower respiratory tract viral infections^1,6^. The mechanisms underpinning seasonality of these infections varies by age and geography^8,14^. A suggestion for future research is to investigate the impact of both age and type of disease on fever healthcare-seeking behaviour, in addition to improving knowledge on aetiology of fever^15^. Fever cases treated at public health facilities are just the tip of the iceberg of the burden within the population^16,17^. Our findings highlight an attributable proportion of fevers treated outside the public sector. While this variation is expected due to factors such as proximity, results suggest the need to increase understanding of behavioural patterns in seeking treatment for fever in countries with a relatively larger private sector^18,19^. This would also enable fair comparisons between public and private sectors in different socio-economic and cultural contexts. In countries with a relatively high burden of fever and where use of the public sector is low, such as in Mozambique and Ethiopia, such an undertaking would be useful for improving case management practises. Currently, the recommended approach is to increase the use of community-based programmes^11,20,21^ in addition to improving physical accessibility.

There were additional data limitations based on the nationally representative survey data used in this study. These include the non-sampling error and a geo-location error introduced to cluster coordinates for personal data protection^22^. The non-sampling error, although unknown, varies at the country-level based on survey quality, which varies by implementing organisation. We also did not explore the influence of seasonality on fever treatment because cross-sectional surveys provide only limited insights into both inter- and intra-annual variability of fever events. This is because such nationally representative household surveys are conducted only every 3–5 years and the duration of surveys is typically two-to-six months. While health facility databases are fragmented and need consolidation by the respective ministries of health, the presence of private health facilities could have impacted on observed uptake of public healthcare facilities^23^. We did not model this competition, since complete spatial databases of private healthcare providers were not available. Moreover, multiple sources of treatment could have been used for a single fever episode^24^. Our findings, however, could be extended to understand other aspects of public healthcare delivery, including estimating the probability of receiving an intervention at a public health facility.

Unlike in previous observational research on treatment practises, the methodology employed here modelled latent characteristics based on the IRT framework^25^. IRT concepts are common to other health applications such as psychometry and could be applied in future household survey analyses to understand the differential item functioning in population sub-groups with similar latent traits^26,27^. We conducted an assessment and generalisation of this methodology to mixed effects regression including gender and socio-economic status^13,28,29^. While inclusion of more regression effects in previous experiments would have been advantageous, the resulting complexity in modelling negatively impacted model performance and efficiency significantly^13^. The strength of this methodology, however, was in producing metrics that can be comparable at the country-level or at the sub-national level and providing insight into public sector service delivery. Moreover, using a Bayesian approach had the added advantage of quantifying uncertainty related to seeking treatment. An additional advantage of this approach was in constraining probability estimates to control for variation in distance to a given health facility.^30^

In conclusion, the accumulation of nationally representative surveys, such as the Demographic Health Surveys, Malaria Indicator Surveys and Multiple Indicator Cluster Surveys provides enormous potential for understanding human health behaviour. This possibility is further increased by the availability of fine spatial resolution population maps as well as georeferenced health facility databases. Health facility databases, however, require more accurate and complete mapping at the national level. The African ministries of health should consider conducting national censuses to establish and update the existing lists assembled by this study. The variation in fever-treatment patterns for children under five estimated here suggests a need to understand healthcare utilisation outside the public sector.

## Methods

### Individual-level data on fever treatment choices

Data on fever treatment choices were assembled from the most recently undertaken nationally representative population-based household surveys in SSA. These were undertaken predominantly in the last five years in most countries and include the Malaria Indicator Survey (MIS) [*n* = 12 surveys], Demographic and Health Survey (DHS) [*n* = 23 surveys], Multiple Indicator Cluster Survey (MICS) [*n* = 6 surveys], the AIDs and Malaria Indicator Survey (AIS) in Mozambique and the 2006 South Sudan Household Survey (SSHS). These data were not available or not georeferenced in 11 countries (Botswana, Cape Verde, Central Africa Republic, Guinea Bissau, Mauritania, Mauritius, Sao Tome and Principe, Somalia, Seychelles, South Sudan, Chad and South Africa). Individual-level data from the most recent household surveys in Djibouti, Equatorial Guinea, The Gambia and Sudan were not available in the public domain, leaving data for 29 countries available for Bayesian analysis. Therefore, countries where georeferenced data were not available were excluded from the analysis of probability of treatment-seeking at public healthcare facilities. Figure 1b was, however, constructed based on a combination of raw counts of fever cases treated from these nationally representative samples at administrative level 1 and population estimates of children under the age of 5 years^31^. To maximise completeness of Fig. 1b, published national reports at administrative level 1 from a recent round of MICS surveys were used in countries where data were not available (in Djibouti, The Gambia, Guinea Bissau, Mauritania, Sao Tome and Principe, Somalia and the one survey in South Sudan). These surveys are internationally standardised and are usually based on a random two-stage cluster sampling design in which clusters are first sampled based on a probability-proportional-to-size basis and thereafter, within each cluster, households are sampled randomly^14,32^. Cluster sizes vary, but were ~15–30 households. Each child’s primary carer or mother was interviewed on child febrile illness two weeks prior to the survey as well as actions undertaken to treat the most recent fever episode. For clarity, treatment-seeking for fever in children under 5 years was based on the carer’s or mother’s response. This study examined individual-level traits related to the indicator on actions taken for treatment of fever in general. Missing data were included as NAs in the Bayesian latent trait estimation if a child had a fever in the period two weeks prior to the survey, but the response on treatment was marked as don’t know.

A well-known phenomenon of fever treatment-seeking behaviour is the decay-effect at a greater travel time or distance from the potential source of treatment^13^. The phenomenon of distance- or travel time-decay has been observed in previous studies^33,34^ and occurs when the usage of health facilities declines with increasing distance in space or time^35,36^. Our methodology for estimating travel times has been documented in several previous studies. In brief, a gridded raster layer of travel time to health facility was estimated by combining, in Geographic Information System (GIS) AccessMod 5^37^, spatial layers on land cover or land use characteristics with roads, elevation and location of health facilities. This spatial modelling approach incorporated elevation to capture the influence of slope on travel speed between different land characteristics. Estimated travel time was included here as an explanatory variable reflecting ability to seek treatment. It is not expected that all febrile patients in very close proximity to a public health facility seek treatment there because some mild-fevers self-resolve, are treated through informal care or private sector health facilities, are home managed or may be treated at a more distant public hospital^24^.

### Spatial databases of health facilities

An inventory of major public-based hospitals in SSA has already been published elsewhere [10.7910/DVN/JTL9VY]^38,39^. In addition to the major regional and district hospitals, spatial databases of lower-tier health facilities (dispensaries, clinics, health posts and health centres) were assembled and georeferenced. The assembled lists consisted mainly of public non-profit health facilities managed by governments, local authorities, faith-based organisations and non-governmental organisations (NGOs). A census of health facilities (public and private) has been conducted in Namibia^40^ and Malawi^41^ and some countries such as Sierra Leone are in the process of conducting Service Availability and Readiness Assessments (SARA)^42^. In other countries such as Kenya, a spatial database of all health facilities was published previously and updated regularly^43^. Spatial health facility databases were incomplete in two provinces of the Democratic Republic of the Congo and in Guinea Bissau. For these countries, travel times to lower-tier facilities were not computed, as shown in Fig. 2b and Fig. 2c. In addition, the probability of seeking treatment for fever was estimated only at major hospitals for these regions. Private or specialised health facilities (e.g., eye clinics, TB centres, special injury units) were excluded from the analysis because; (a) a complete list of private health facilities was not available in these countries and (b) the specialised health facilities, in general, do not treat uncomplicated febrile illness.

### Modelled probability of seeking fever treatment

A unidimensional three-parameter model (3PL) based on Item Response Theory (IRT)^44^ was used to estimate latent individual traits for actions undertaken to treat a fever episode. Analysis was performed separately for each country. Travel time to nearest health facility and place of residence were extracted based on our methods demonstrated in Refs ^13,45^ for Namibia and replicated for all the counties in SSA (Fig. 2a–c). This variable as well as residence (urban or rural status) was used as an explanatory variable for the individual ability parameter within a hierarchical Markov chain Monte Carlo (MCMC) Bayesian framework. This methodology has also been evaluated elsewhere including the effect of residence (urban and rural)^13^. The probability of seeking treatment for fever was modelled via:

1 $$P\left( {Y_{ij} = 1|\theta _j,a_i,b_i,c_i} \right) = c_i + \left( {1 - c_i} \right)\frac{{\exp \left\{ {\mathop {\sum}\limits_{k = 1}^m {\left( {a_{ik}\theta _{jk} - b_{ik}} \right)} } \right\}}}{{\left[ {1 + \exp \left\{ {\mathop {\sum}\limits_{k = 1}^m {\left( {a_{ik}\theta _{jk} - b_{ik}} \right)} } \right\}} \right]}}$$

Where *Y*_*ij*_ represents a dichotomous behavioural response (0 or 1) if treatment was sought for an individual *j*(*j*=1,……,*N*) and *i*=1 on use of public healthcare for treatment. *f*(*θ*,*a*,*b*,*c*) is a collection of unknown parameters with *f*(*θ*,*a*,*b*,*c*)=*f*(*θ*) *f*(*a*) *f*(*b*) *f*(*c*) as the posterior density we aim to evaluate. In general, *θ*_*j*_=*θ*_*j*1_……,*θ*_*jk*_,……*θ*_*jm*_; −∞<*θ*_*jk*_>+∞ for *k*=1,……*m* dimensions (although only one dimension was considered) represents the person trait (i.e., individual-level ability) and has a central influence on discriminant and difficulty parameters. This was parameterised as *θ*_*j*_=*α*_*j*_+*β*_1*j*_X_1*j*_+*β*_2*j*_X_2*j*_ with *β*_*j*_ representing coefficients of dependent variables *X*_*j*_ exploring differences in ability. The primary focus was on the *θ*_*j*_ parameter, rather than the coefficients. Travel time and residence (urban or rural) were included as predictor variables on the ability parameter. This study did not include all the possible socio-demographic variables due to the marginal computational gains from doing so, as demonstrated in previous assessments of this methodology^13^. *a*_*ik*_ in Equation 1 represents the item discrimination parameters, and is positive (*a*_*ik*_>0), for different types of treatment centres or health facilities used (Hospital, dispensary or health centre); *b*_*ik*_ (−∞<*b*_*ik*_<∞) represents survey item difficulty parameters, while *c*_*i*_ (0<*c*_*i*_<1) represents the threshold (i.e., minimum) probability parameter for individual *i*. The probability threshold parameter and the ability parameter are most relevant for the current health application. Given equation 1 applies to one item (*i*=1 and *k*=1) the discrimination and difficulty parameters have a marginal influence on the overall probability. The probability threshold parameter was constrained based on a prior distribution at greater than zero and less than one. The posterior distribution is then expressed as the product of the likelihood and prior distributions for unknown parameters as:

2 $$f\left( {\theta ,a,b,c|y} \right)\begin{array}{*{20}{c}} { \propto \begin{array}{*{20}{c}} {L\left( {y|\theta ,a,b,c} \right)f\left( {\theta ,a,b,c} \right)} \end{array}} \end{array}$$

Bayesian implementation was completed by assigning prior distributions to parameters *a*_*i*_>0, $a_i\sim N\left( {\mu _a,\sigma _a^2} \right)I\left( {a_i > 0} \right)$ half normal and $I\left( \cdot \right)$ is an indicator function; *c*∈(0,1] based on a beta distribution and a normal prior for *b*_*i*_,*α*,*β*. Model implementation was evaluated using a combination of Gelman–Rubin with Raftery–Lewis diagnostic to check for convergence^46,47^ using three chains in JAGS version 4.2.0 and the rjags package in R version 3.3.1^48^. The sample IRT code is included in the Supplementary Software 1. In brief, 550,000 iterations were implemented with a burn in of 50,000 and a thinning factor of 500, resulting in a weakly dependent sample size of *n* = 1000. An example of convergence diagnostics is shown in Supplementary Figure 2 and Supplementary Figure 3. Posterior summaries of the parameters were used to back-calculate the probability of seeking treatment for fever (via Equation 1) and the response curve was plotted by travel time. Country-level summaries were also produced for each type of health facility. Validation was checked using a 10% subset dataset not included in the main analysis in the 29 countries separately. This validation set was selected randomly from each nationally representative survey. The remaining 90% of data were used for the main analysis. The model was then applied to the validation set and the predicted probability of seeking treatment for fever transformed to a binary outcome. Model accuracy was evaluated based on the misclassification error of the binary outcomes. Lastly, the receiver operating characteristic (ROC) was used to estimate the specificity and sensitivity of predictions when compared to observed responses from survey data. Results of the model validation exercise are included in the Supplementary Data 2.

### Code availability

Relevant IRT code is provided in the Supplementary Software 1.

## Electronic supplementary material

Supplementary Information

Description of Additional Supplementary Files

Supplementary Data 1

Supplementary Data 2

Supplementary Software 1

## Data Availability

Nationally representative household survey data for the countries are available in the public domain at http://dhsprogram.com/data/available-datasets.cfm. Major hospital data are available in a generalist repository and cited within the manuscript: [10.7910/DVN/JTL9VY]. For complete health facility databases, the authors are working with the World Health Organisation to make these data publically available through the African Health Observatory where these data will be hosted. The raster layers generated during and/or analysed during the current study (i.e., travel time to health facilities and the probability of seeking treatment) are available from generalist figshare repository: 10.6084/m9.figshare.7160363.
