# Supplementary material for: National and sub-national variation in patterns of febrile case management in sub-Saharan Africa
Source: Nat Commun. 2018 Nov 26;9:4994. doi: 10.1038/s41467-018-07536-9 (PMC6255762; doi:10.1038/s41467-018-07536-9)
Supplement: Supplementary file 1 — Supplementary Information [file 41467_2018_7536_MOESM1_ESM.pdf]

Supplementary Information

National and sub-national variation in patterns of febrile case management in sub-Saharan Africa

Victor A. Alegana*, Joseph Maina, Paul O Ouma, Peter M Macharia, Jim Wright, Peter M. Atkinson, Emelda A. Okiro, Robert W. Snow, Andrew J. Tatem

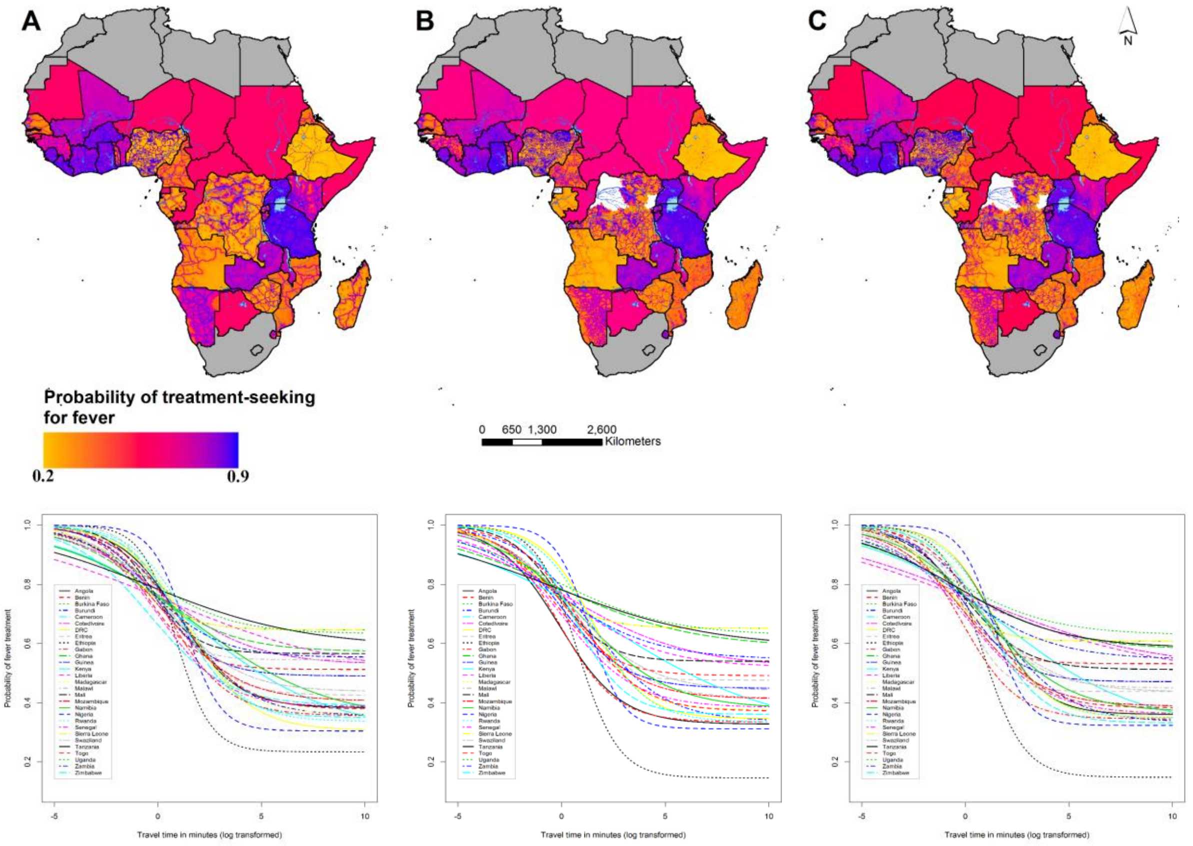

Supplementary Figure 1: National and sub-national variation in probability of fever treatment (posterior median maps and plots) at country level in sub-Saharan Africa (*n*=29 countries) by facility type **A)** hospitals, **B)** health centres, and **C)** dispensaries or clinics or health posts. The probability was modelled based on the 3PL model from nationally-representative household surveys. Hospitals, in general, had a longer lag travel-time before decay compared to other health facility types suggesting a shorter distance decay to dispensaries or clinics or health posts. Moreover, the use of primary health facilities in the public sector for fever treatment was slightly higher compared to major hospitals where the probability of treatment was similar to the national posterior median.

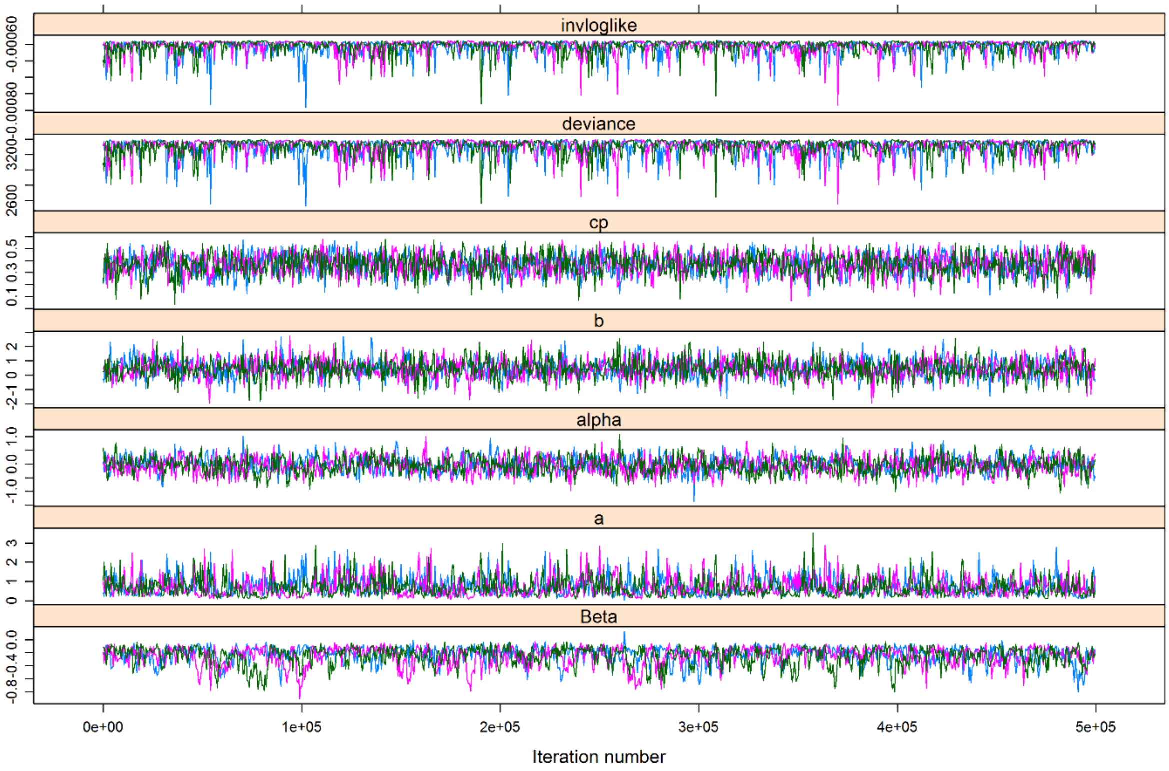

Supplementary **Figure 2**: Convergence diagnostic: Example of trace plots extracted for Bayesian IRT model for Ghana for the monitored parameters over the duration of model run.

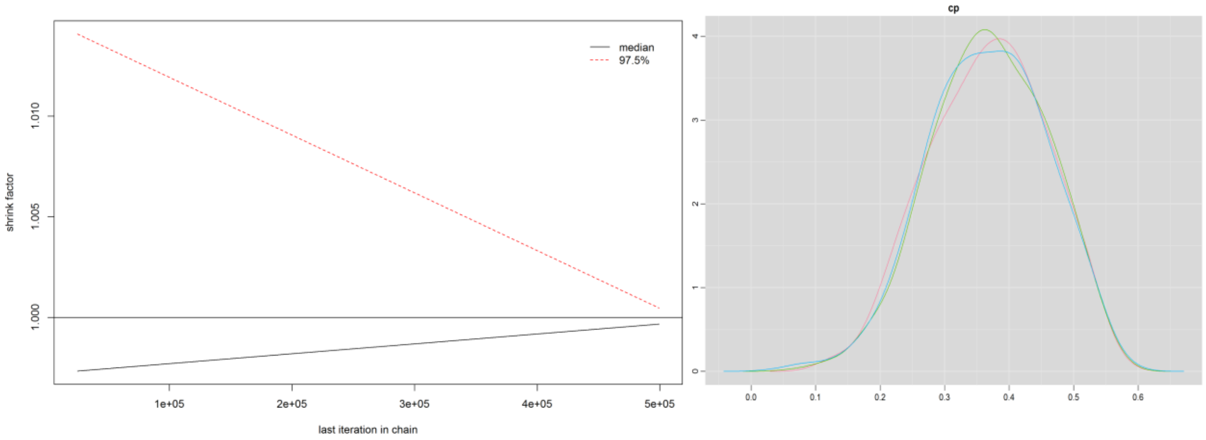

Supplementary **Figure 3**: Gelman plot for the probability parameter for Ghana data and the density plot for the MCMC samples (3 chains) for the same parameter.
