# Supplementary material for: National and sub-national variation in patterns of febrile case management in sub-Saharan Africa
Source: Nat Commun. 2018 Nov 26;9:4994. doi: 10.1038/s41467-018-07536-9 (PMC6255762; doi:10.1038/s41467-018-07536-9)
Supplement: Supplementary file 2 — Description of Additional Supplementary Files [file 41467_2018_7536_MOESM2_ESM.pdf]

Description of Additional Supplementary Files

File Name: Supplementary Data 1

Description: Summary of assembled nationally representative survey data. All household survey data used in the study were obtained from DHS program (https://dhsprogram.com/data/), obtained via online application and registration.

File Name: Supplementary Data 2

Description: Country level modelled estimates of probability of treatment at population level (Posterior median and the 95% Bayesian credible interval) by travel time and by facility type.

File Name: Supplementary Software 1

Description: A generalised R statistical software computer code for Bayesian three parameter estimation in IRT with implementation is conducted via MCMC (R-JAGS). Also includes code for reproducing IRT curves (Figure 3A and graphs in Supplementary Figure 1).
