# Supplementary material for: National and sub-national variation in patterns of febrile case management in sub-Saharan Africa
Source: Nat Commun. 2018 Nov 26;9:4994. doi: 10.1038/s41467-018-07536-9 (PMC6255762; doi:10.1038/s41467-018-07536-9)
Supplement: Supplementary file 5 — Supplementary Software 1 [file 41467_2018_7536_MOESM5_ESM.pdf]

Supplementary software 1

National and sub-national variation in patterns of febrile case management in sub-Saharan Africa

Victor A. Alegana, Joseph Maina, Paul O Ouma, Peter M Macharia, Jim Wright, Peter M. Atkinson, Emelda A. Okiro, Robert W. Snow, Andrew J. Tatem

Computer Code Item Response Theory (IRT)

This is a generalised code for three parameter estimation in IRT. Section 1 of the code is a generalisation for a sample size of n in k dimensions. Implemetation is done via MCMC (R-JAGS) monitoring the three parameters of interest. Section 2 is the reproduction of IRT curves in R statistical software that generated Figure 3A and graphs in Supplementary Figure 1.

```
## Section 1
## Generalised three-parameter item response model main R-Source code for JAGS
## Three parameters as a, b, c
## Monitor the three parameters for MCMC implementation to ensure convergence
## N is sample size while k is dimension, e.g. k=1, 2…

model
{
  for (i in 1:N) {
      for (k in 1:K) {
                    p[i,k] <- c[k]+((1-c[k])*(exp(a[k]*(theta[i]-b[k]))/(1+exp(a[k]*(theta[i]-b[k])))))
                    Y[i,k] ~ dbern(p[i,k])
       }

       theta[i] ~ dnorm(0,1)
   }

   for (k in 1:K) {
      a[k] ~ dnorm(0,1)I(0,)
      b[k] ~ dnorm(0,1)
      c[k] ~ dbeta(b11,b12)
   }

b11 ~ dnorm(1,100)
b12 ~ dnorm(1,100)
}

## relevant code for plotting the three parameters and probability estimates in R
## Define three input parameters and probability

probfun <- function(a.param, b.param, c.param, theta) { #reads vector of parameters
  prob<- matrix(0,length(theta),length(a.param))
  for (i in 1:length(theta)) { # iterate over length of the vector
              # compute output as
```

```
                            prob[i,] <- c.param +((1-c.param) / (1+(exp(a.param*theta[i] - b.param)))) #output
  return(prob)
}

## section 2
## code to plot response curves
out <- probfun(param$a_Median, param$b_Median, param$cp_Median,theta)
a.med.unique <- unique(param$a.med)
cols <- seq(1,60,1)
lt <- seq(1,60,1)
lnames <- as.character(param$Country)
lnames
for (i in 1:length(a.med)){
  #if (i==1)plot(theta, out[,i], type="p", xlim=c(-5,10), ylim=c(0.1,1), pch=19, col = "grey")
  #if (i>1)lines(theta, out[,i], type="p", xlim=c(-5,10), ylim=c(0.1,1), pch=19, col = "grey")

 if (i==1)plot(theta, out[,i], type="l",
              xlim=c(-5,10),
              ylim=c(0.1,1),
              col = cols[i], lwd = 2, lty=lt[i],
              ylab="Probability of seeking fever treatment", xlab="Travel time in minutes (log transformed)")
 if (i>1)lines(theta, out[,i], type="l",
              xlim=c(-5,10),
              ylim=c(0.1,1),
              col = cols[i], lwd = 2, lty=lt[i])

  #axis(1, at=theta,labels=round(xlables, digits=1),cex=1, col.axis="black ", las=1, cex.axis=1,tck=-0.01)
}
rect(xleft=2.302585, xright=4.787492, ybottom=0,ytop =2, col= rgb(1,0,0,alpha=0.5))
abline(v= c(2.302585, v=3.401197,v=4.094345, v=4.787492), lty=1, lwd=1, col="red")
grid(lwd = 2)
  legend(x=-5,y=0.8,
       legend=lnames,
       col=seq(1,60,1),
       lty=seq(1,60,1),
       cex=0.75,
       box.col="dark grey",
       box.lwd=1,
       #family='times roman',
       lwd=2)

  ## End of code
```
